# Chronic Hepatitis B Virus Infection and Risk of Stroke Types: A Prospective Cohort Study of 500 000 Chinese Adults

**DOI:** 10.1161/STROKEAHA.123.043327

**Published:** 2023-11-09

**Authors:** Elizabeth M. Hamilton, Ling Yang, Neil Wright, Iain Turnbull, Alexander J. Mentzer, Philippa C. Matthews, Yiping Chen, Huaidong Du, Christiana Kartsonaki, Yuanjie Pang, Pei Pei, Huizi Tian, Xiaoming Yang, Daniel Avery, Canqing Yu, Jun Lv, Robert Clarke, Liming Li, Iona Y. Millwood, Zhengming Chen

**Affiliations:** 1 Clinical Trial Service Unit and Epidemiological Studies Unit (E.M.H., L.Y., N.W., I.T., Y.C., H.D., C.K., X.Y., D.A., R.C., I.Y.M., Z.C.), Nuffield Department of Population Health, University of Oxford, United Kingdom.; 2 Medical Research Council Population Health Research Unit (L.Y., C.K., I.Y.M., Z.C), Nuffield Department of Population Health, University of Oxford, United Kingdom.; 3 Wellcome Centre for Human Genetics (A.J.M.), University of Oxford, United Kingdom.; 4 Division of Infection and Immunity, University College London, United Kingdom (P.C.M.).; 5 Matthews lab HBV Elimination Laboratory, The Francis Crick Institute, London, United Kingdom (P.C.M.).; 6 Department of Epidemiology and Biostatistics, School of Public Health, Peking University Health Science Center, Beijing, China (Y.P., C.Y., J.L., L.L.).; 7 Peking University Center for Public Health and Epidemic Preparedness and Response, Beijing, China (Y.P., P.P., C.Y., J.L., L.L.).; 8 Non-Communicable Diseases Prevention and Control Department, Henan, China (H.T.).

**Keywords:** cerebral hemorrhage, cohort studies, incidence, ischemic stroke, premature mortality, stroke

## Abstract

**BACKGROUND:**

Stroke is a leading cause of mortality and permanent disability in China, with large and unexplained geographic variations in rates of different stroke types. Chronic hepatitis B virus infection is prevalent among Chinese adults and may play a role in stroke cause.

**METHODS:**

The prospective China Kadoorie Biobank included >500 000 adults aged 30 to 79 years who were recruited from 10 (5 urban and 5 rural) geographically diverse areas of China from 2004 to 2008, with determination of hepatitis B surface antigen (HBsAg) positivity at baseline. During 11 years of follow-up, a total of 59 117 incident stroke cases occurred, including 11 318 intracerebral hemorrhage (ICH), 49 971 ischemic stroke, 995 subarachnoid hemorrhage, and 3036 other/unspecified stroke. Cox regression models were used to estimate adjusted hazard ratios (HRs) for risk of stroke types associated with HBsAg positivity. In a subset of 17 833 participants, liver enzymes and lipids levels were measured and compared by HBsAg status.

**RESULTS:**

Overall, 3.0% of participants were positive for HBsAg. HBsAg positivity was associated with an increased risk of ICH (adjusted HR, 1.29 [95% CI, 1.16–1.44]), similarly for fatal (n=5982; adjusted HR, 1.36 [95% CI, 1.16–1.59]) and nonfatal (n=5336; adjusted HR, 1.23 [95% CI, 1.06–1.44]) ICH. There were no significant associations of HBsAg positivity with risks of ischemic stroke (adjusted HR, 0.97 [95% CI, 0.92–1.03]), subarachnoid hemorrhage (adjusted HR, 0.87 [95% CI, 0.57–1.33]), or other/unspecified stroke (adjusted HR, 1.12 [95% CI, 0.89–1.42]). Compared with HBsAg-negative counterparts, HBsAg-positive individuals had lower lipid and albumin levels and higher liver enzyme levels. After adjustment for liver enzymes and albumin, the association with ICH from HBsAg positivity attenuated to 1.15 (0.90–1.48), suggesting possible mediation by abnormal liver function.

**CONCLUSIONS:**

Among Chinese adults, chronic hepatitis B virus infection is associated with an increased risk of ICH but not other stroke types, which may be mediated through liver dysfunction and altered lipid metabolism.

Stroke is a major cause of premature mortality and permanent disability worldwide, with particularly high rates in China, with 3.9 million incident cases and 2.2 million deaths in 2019.^1^ Compared with Western populations, China has a relatively high proportion of intracerebral hemorrhage (ICH), accounting for an equal number of deaths to those from ischemic stroke (IS), despite IS having a 5-fold higher incidence compared with ICH.^1,2^ Although several major risk factors for stroke are established, they do not explain the large difference in stroke rates between China and Western populations and between regions within China.^3,4^ Identification of new risk factors for stroke and stroke types, including chronic infections, is needed, which may inform prevention and treatment.

Chronic hepatitis B virus (HBV) affects >250 million people worldwide and is responsible for 0.8 million deaths per year, predominantly from chronic liver disease and liver cancer.^5^ China accounts for 1 in 3 global cases of chronic HBV,^6^ where the major route of transmission is mother-to-child transmission.^7^ Over 90% of unvaccinated neonates and 50% of children aged under 5 years exposed to HBV infection will develop chronic infection, compared with <5% among adults.^8^ Chronic HBV infection can cause liver damage and alter the biosynthetic and metabolic capacity of the liver, impacting coagulation status^9^ and lipid levels,^10–12^ which may also contribute to the risk of nonliver diseases.

Previous cohort studies of national health insurance data in Taiwan have reported a positive association between liver cirrhosis and ICH.^13–15^ There were also reports of associations of HBV infection with risks of stroke in Korean,^16^ Taiwanese,^17,18^ and Western populations.^11,19^ However, several of these studies are limited by their cross-sectional nature, modest size, noncommunity settings, having coinfection with other blood-borne viruses, or lacking information on stroke types.^11,18,19^ To date, no prospective evidence is available from mainland China, which has a high burden of both chronic HBV infection and stroke.

Therefore, in the prospective China Kadoorie Biobank of 0.5 million Chinese adults, we aimed to (1) assess the associations of hepatitis B surface antigen (HBsAg) positivity with risks of stroke types, including ICH, IS, and subarachnoid hemorrhage and (2) explore the association between HBsAg positivity and a range of metabolic traits including blood biochemistry.

## METHODS

### Study Population

This article follows the Strengthening the Reporting of Observational Studies in Epidemiology statement for cohort studies (Supplemental Material). The China Kadoorie Biobank Study design included a baseline survey conducted from 2004 to 2008 among 512 726 men and women aged 30 to 79 years, from 5 urban and 5 rural areas.^20^ Trained health workers used a laptop-based questionnaire to collect information about sociodemographic and lifestyle factors, and medical conditions diagnosed by a doctor, including liver cancer, cirrhosis, or chronic viral hepatitis. Physical measurements were undertaken, including blood pressure, height, weight, and a nonfasting blood sample for on-site tests (including HBsAg) and long-term storage. Resurveys were conducted in 2008, 2013 to 2014, and 2020 to 2021 among a subset (4%–5%) of participants, with additional measurements including carotid intimal thickness and carotid plaque score conducted in 2013 to 2014 resurvey (Supplemental Methods).^21,22^

All participants provided written informed consent, and international, national, and regional ethics approvals were obtained. The data used in this study are available to the wider research community, which can be applied through the China Kadoorie Biobank website (www.ckbiobank.org).

### Measurement of Chronic HBV Infection

HBsAg was measured in participants at baseline using a point of care lateral flow rapid diagnostic test (ACON dipstick) applied to venous whole blood, interpreted as positive, negative, or unclear. Participants were classified as having chronic HBV infection if their HBsAg test was positive.^20,23^

### Follow-Up for Stroke Outcomes

The vital status of participants and disease events occurring during follow-up were monitored via electronic linkage to mortality and morbidity registries (Supplemental Methods). Information on hospitalization episodes was collected via linkage to national health insurance systems, which has almost universal coverage in study areas. The outcomes were *International Classification of Diseases-Tenth Revision* (*ICD-10*) coded by trained health workers who were blinded to baseline participant information. By January 1, 2018, 48 167 (9.6%) participants died and 5226 (1.0%) were lost to follow-up.

Stroke end points assessed included ICH (*ICD-10* I61), IS (I63), subarachnoid hemorrhage (I60), other/unspecified stroke (I64), and total stroke (ICD-10 160, 161, 163, 164). Fatal strokes were defined as one with any death within 30 days and a nonfatal event denoted survival at least 30 days after stroke diagnosis. To ensure the accuracy of stroke reporting, adjudication of stroke occurrence was undertaken by a review of retrieved original medical records by a panel of clinical specialists in China.^24^

### Measurement of Blood Biochemistry

As part of a nested cardiovascular case-control study, blood biochemistry measurements were made in baseline plasma samples in a subset of up to 17 833 participants.^25^ This included 5429 and 4758 incident cases of IS and ICH, respectively, with a censoring date of January 1, 2015. Controls (n=6223) were selected among those who were free of diagnosis of cardiovascular disease (CVD) at the censoring date, frequency matched by age, sex, and area. Cases and controls had no prior history of CVD, cancer, or lipid-lowering treatment reported at baseline. Plasma biochemistry measurements were conducted at the accredited Wolfson Laboratory, Oxford,^20^ where lipid concentrations (total cholesterol, low-density lipoprotein-cholesterol, high-density lipoprotein-cholesterol, and triglycerides), liver enzymes (ALT [alanine transaminase], AST [aspartate aminotransferase], and GGT [gamma-glutamyl transferase]), albumin, fibrinogen, vitamin D, CRP (C-reactive protein), creatinine, uric acid and vitamin D were measured using AU680 Chemistry Analysers (Beckman Coulter). Abnormal liver test levels were defined as ALT>30 μmol/L, AST>30 μmol/L, GGT>50 μmol/L, and albumin<35 g/L.^26^

### Statistical Analyses

Individuals with missing (n=8194) or unclear HBsAg status (n=3539) were excluded from the analysis, in addition to 2 people with missing body mass index (BMI) data, leaving 500 991 people in the main analysis (Figure S1). Baseline characteristics of participants with missing compared with nonmissing and unclear data are shown in Table S1, where missing data occurred due to a lack of HBsAg test supplies in 2 rural sites in the first few months of the study.

Baseline characteristics of participants by HBsAg status were standardized by sex, age (5-year groups), and study site (10 sites). The number of events and incidence rate per 100 000 person-years at risk standardized by age and sex were calculated. Cumulative incidence curves of stroke types by HBsAg status were constructed using the cuminc function in R, where all-cause mortality was treated as a competing risk, and age attained on the study was the timescale. The Gray test (a modified χ^2^ test) was used to compare cumulative incidence by HBsAg status.

Cox proportional hazards models were used to estimate hazard ratios (HRs) and 95% CI for stroke associated with HBsAg positivity, with age at risk as the timescale. Analyses were stratified by baseline hazard among sex, age-at-risk (5-year groups), and study site (10 sites) and adjusted for education (no formal education, primary/middle school, and high school or above) and household income (<5000, 5000–19 999, and >20 000 yuan), smoking (never regular and ever regular), alcohol (never, occasional, and regular), physical activity (metabolic equivalent of task-hours/day), dietary factors (regular [≥4× per week] or lower intake of fresh fruit, dairy, and meat intake), BMI (kg/m^2^), systolic blood pressure (mm Hg), and history of diabetes or cancer. For ICH and IS, HRs for fatal and nonfatal stroke were also calculated, with tests of heterogeneity by fatality status performed. The proportional hazards assumption was assessed using the test of proportionality and visualizing Schoenfeld residuals, with no evidence of violation of the Cox proportional hazards assumption (Table S2; Figure S2).

If HRs were statistically significant, estimates of absolute risk were additionally calculated. This included the attributable fraction, which is the proportion of stroke cases due to HBsAg positivity among the exposed (ie, among those with chronic HBV),^27^ using the formula: $AF=\frac{HR-1}{HR}$, and the population attributable fraction, which is the proportion of stroke cases in the total population due to HBsAg positivity, using Miettinen formula: $PAF=P_{c}\frac{\left(HR-1\right)}{HR}$, where *P*_c_ is the prevalence of HBV among stroke cases.^28^ The number of stroke cases attributed to chronic HBV in the Chinese population was estimated by multiplying the population-attributable fraction with reported Chinese stroke prevalence estimates from the 2019 Global Burden of Disease Study.^29^ Subgroup analyses with tests of heterogeneity for categorical variables and trend for ordered categorical variables were performed to examine potential effect modification of associations by sex, age, urban/rural location, education, income, smoking status, alcohol intake, BMI category, systolic blood pressure, prior history of diabetes, or CVD. Tests of trend were performed using a χ^2^ test for 1 degree of freedom, with the null hypothesis of no linear trend.

Sensitivity analyses were undertaken among (1) adjudicated stroke cases only; (2) excluding participants with baseline chronic liver disease, CVD, or cancer; (3) excluding first 2 years of study follow-up; and (4) restricting analyses to first stroke of any type. Participants with prior CVD were retained in the main analysis, as, consistent with the epidemiology of chronic HBV in China, it was assumed most participants acquired chronic HBV via mother-to-child transmission.^7^

For BMI, systolic blood pressure, carotid intimal thickness, and blood biochemistry, multivariable linear regression was used to calculate weighted marginal mean values by HBsAg status. For participants in the CVD case-control study, inverse probability-weighted linear regression was used to adjust for case ascertainment as previously described (Supplemental Methods).^30^ We constructed a liver test abnormality score incorporating abnormal transaminases and albumin, where a maximum of 1 point was awarded for the presence of abnormal transaminase (ALT or AST>30 μmol/L) and 1 point for albumin <35 g/L. Logistic regression was used to estimate adjusted odds ratios (ORs) for stroke stratified by abnormal liver enzymes, hypoalbuminemia, and liver test abnormality score. Mediation of the HBV-stroke association by liver enzymes, albumin, and lipids was evaluated with a likelihood ratio test that assessed the change in likelihood ratio χ^2^ comparing models additionally adjusted for liver enzymes, albumin, and lipids.

Analyses used R software, Version 4.0.2.

## RESULTS

Among 500 991 participants, the mean age was 52.1 (SD, 10.7) years, 41.0% were men, 55.4% lived in a rural area, and 18.2% received no formal education (Table 1). The overall HBsAg prevalence was 3.0% (men, 3.4%; women, 2.8%), decreasing with age and higher in urban areas (Figure S3). Compared with HBsAg-negative people, HBsAg-positive participants were younger, more likely to be men, had lower education and income levels, drank less alcohol (among men), and had a higher proportion of baseline chronic liver disease (Table 1). These associations were similar in the nested case-control CVD subset and second resurvey (Tables S3 and S4).

**Table 1.**
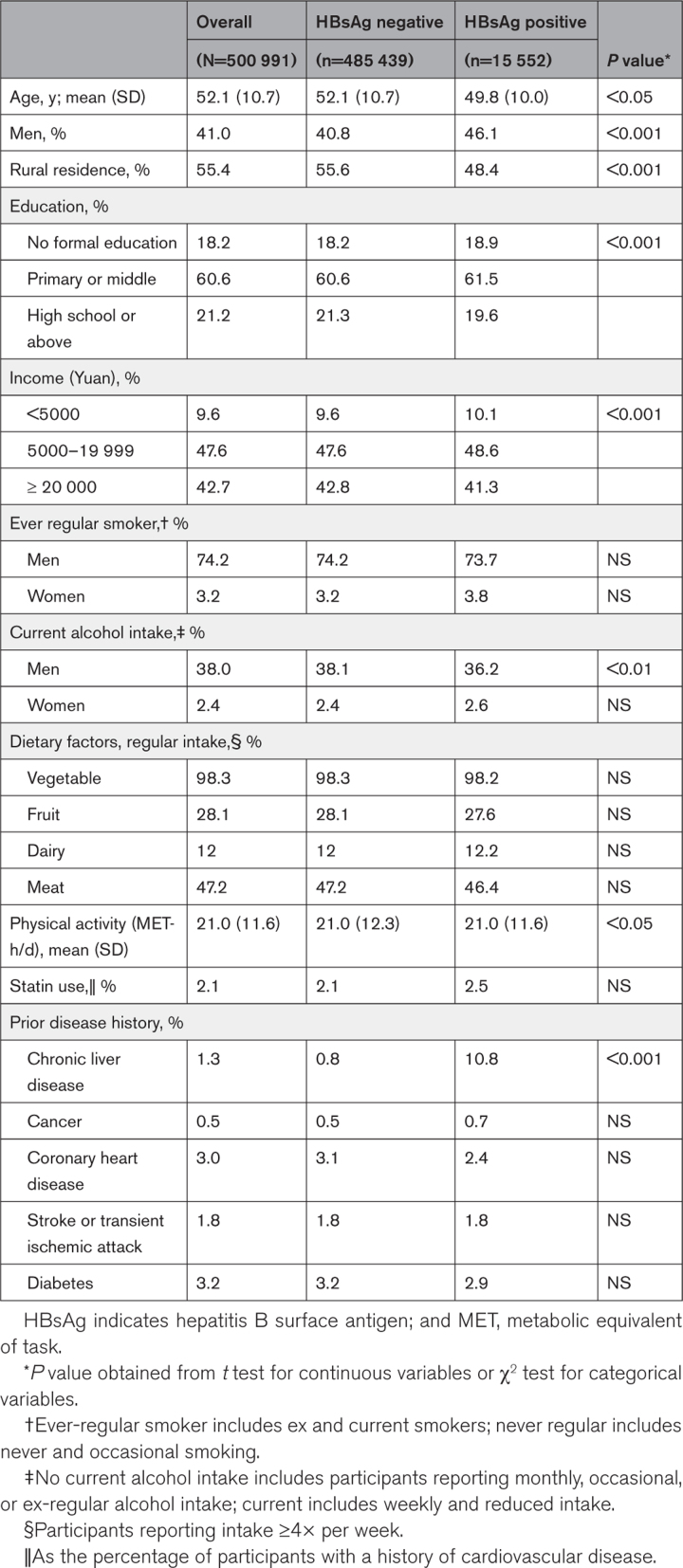
Baseline Characteristics of Participants by HBsAg Status

**Figure 1.**
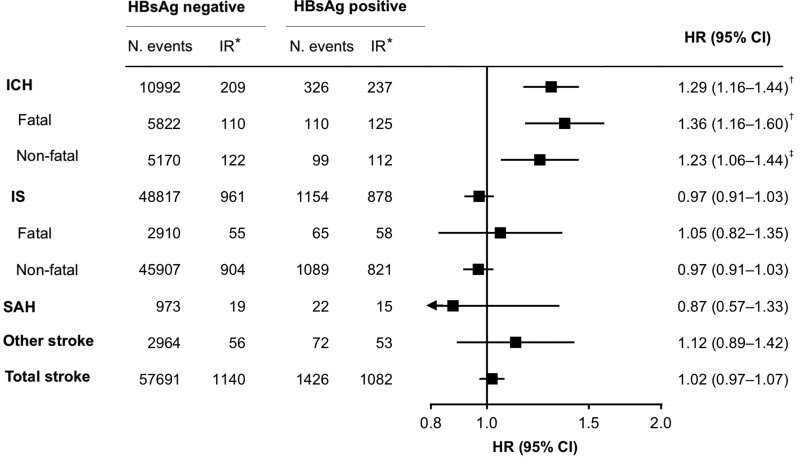
**Number of events, incidence rate of stroke types, and adjusted hazard ratios (HRs) for stroke types by hepatitis B surface antigen (HBsAg) status.** HRs are stratified by age-at-risk, sex, study site and adjusted for education, household income, smoking status, alcohol intake, physical activity, dietary factors, body mass index (BMI), systolic blood pressure, and baseline diabetes or cancer. ICH indicates intracerebral hemorrhage; IR, incidence rate; IS, ischemic stroke; PYAR, person-years at risk; and SAH, subarachnoid hemorrhage. *Age and sex standardized incidence rate per 100 000 PYAR. †*P*<0.01. ‡*P*<0.001.

During a mean follow-up of 10.8 years, 59 117 incident strokes occurred, including 49 971 IS, 11 318 ICH, 995 subarachnoid hemorrhage, and 3036 other/unspecified strokes. Of incident strokes, about 90% were confirmed by computed tomography/magnetic resonance imaging. Incidence rates of ICH were higher among HBsAg-positive participants (237 per 100 000) compared with negative counterparts (209 per 100 000), while they were higher in HBsAg-negative participants for all other stroke types (Figure 1). By age 75 years, 4.8% of HBsAg-positive compared with 4.4% of HBsAg-negative participants had an ICH (*P*<0.01), while, for IS, 18% positive compared with 19% negative participants had events (*P*<0.01), and there was no significant difference in cumulative incidences for other stroke types (Table S5; Figure S4).

**Figure 2.**
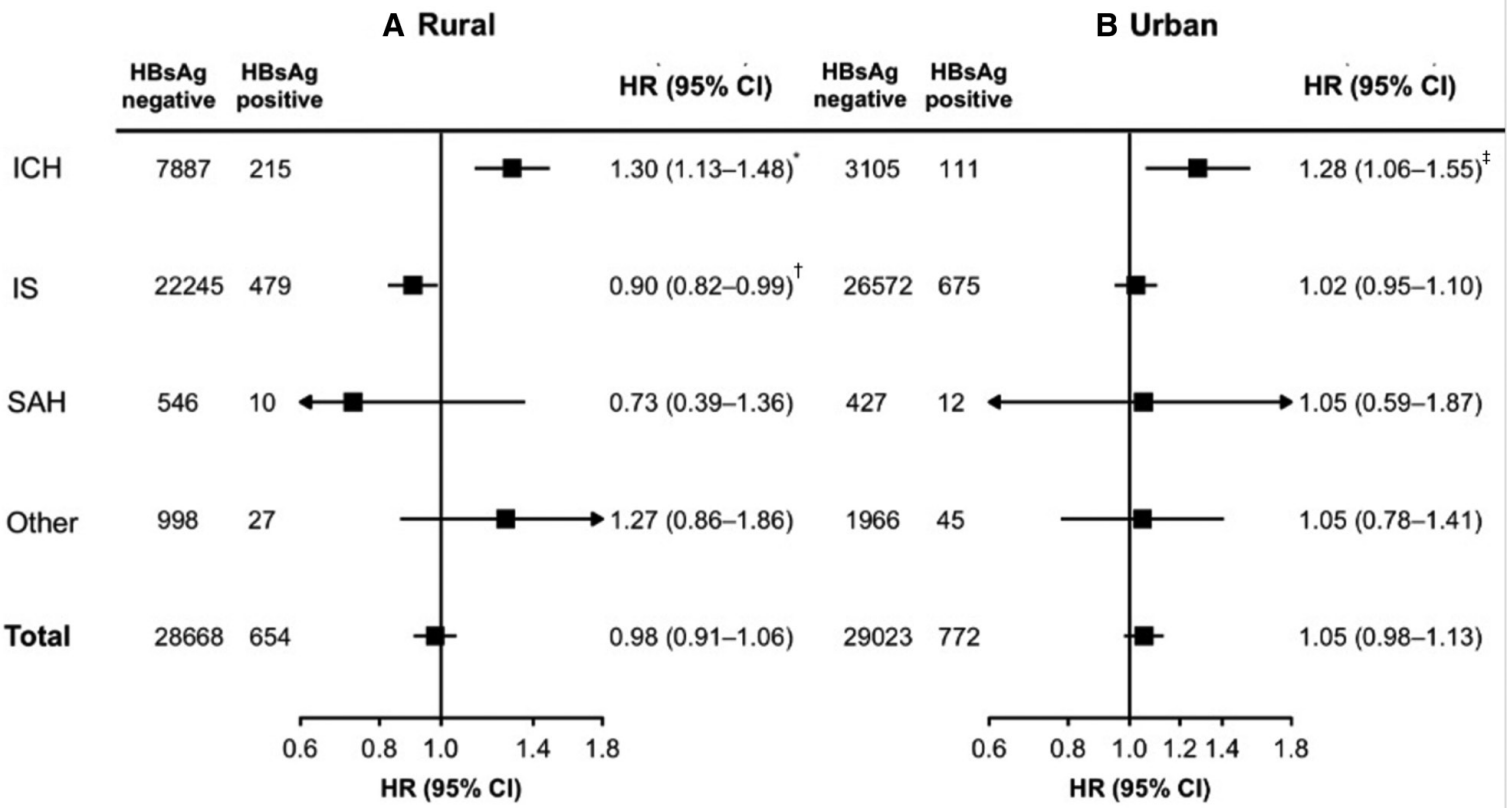
**Adjusted hazard ratios (HRs) for stroke and hepatitis B surface antigen (HBsAg) positivity among rural and urban participants.** HRs are stratified by age-at-risk, sex, study site and adjusted for education, household income, smoking status, alcohol intake, physical activity, dietary factors, body mass index (BMI), systolic blood pressure, and baseline diabetes or cancer. ICH includes intracerebral hemorrhage; IS, ischemic stroke; and SAH, subarachnoid hemorrhage. *P<0.05. †*P*<0.01. ‡*P*<0.001.

After multivariable adjustment, compared with HBsAg-negative participants, HBsAg-positive individuals had HRs of 1.29 (95% CI, 1.16–1.44) for ICH, 0.97 (95% CI, 0.91–1.03) for IS, 0.87 (95% CI, 0.57–1.33) for subarachnoid hemorrhage, 1.12 (95% CI, 0.89–1.42) for other/unspecified stroke, and 1.02 (95% CI, 0.97–1.07) for total stroke (Figure 1), which were similar for participants in rural and urban areas (Figure 2). For ICH, a higher risk was found among HBsAg-positive individuals for both fatal (n=5982 cases; HR, 1.36 [1.16–1.59]) and nonfatal (n=5336; 1.23 [1.06–1.44]) stroke events. For IS, no statistically significant change in risk was found for either fatal (n=2975; 1.05 [0.82–1.35]) or nonfatal (n=46 996; 0.97 [0.91–1.03]) events. While there was significant statistical heterogeneity between HRs for risk of ICH and IS (*P*_heterogeneity_<0.001), within ICH and IS separately, there was no heterogeneity between fatal and nonfatal strokes (ICH: *P*_heterogeneity_=0.75; IS: *P*_heterogeneity_=0.51).

Similar results were found when confining the analyses to adjudicated stroke cases, with adjusted HRs of 1.29 (1.07–1.55) for ICH (n=3768) and 0.95 (0.87–1.04) for IS (n=21 181; Table S6). For ICH, the estimated HR associated with HBsAg positivity was greater among ever-regular smokers than never-regular smokers (HR, 1.46 [95% CI, 1.25–1.71] versus 1.16 [95% CI, 0.99–1.35]; *P*_heterogeneity_=0.04) but did not significantly differ between other population subgroups (Figure S5) or by study site (Tables S7 and S8). Excluding participants with baseline disease, first 2 years of follow-up, or restricting the analysis to the first stroke did not materially alter HRs (Tables S9 through S11).

The attributable fraction of overall ICH events among HBsAg-positive participants was 25% (31% for fatal ICH and 22% for nonfatal ICH). The population attributable fraction for ICH events was 0.7%, meaning that of 2.46 million ICH cases in 2019^29^; ≈30 000 ICH events were attributed to chronic HBV in Chinese adults.

Compared with HBsAg-negative people, HBsAg-positive participants had lower mean BMI (23.5 versus 23.7 kg/m^2^), systolic blood pressure (130.5 versus 131.2 mm Hg), and carotid intimal thickness but no difference in carotid plaque score (Table 2). Among the biochemistry subset, HBsAg-positive participants had lower total cholesterol, low-density lipoprotein-cholesterol, triglyceride, and albumin and higher ALT, AST, GGT, and vitamin D but no difference in high-density lipoprotein-cholesterol, fibrinogen, CRP, glucose, uric acid, or creatinine compared with HBsAg-negative individuals (Table 2).

**Table 2.**
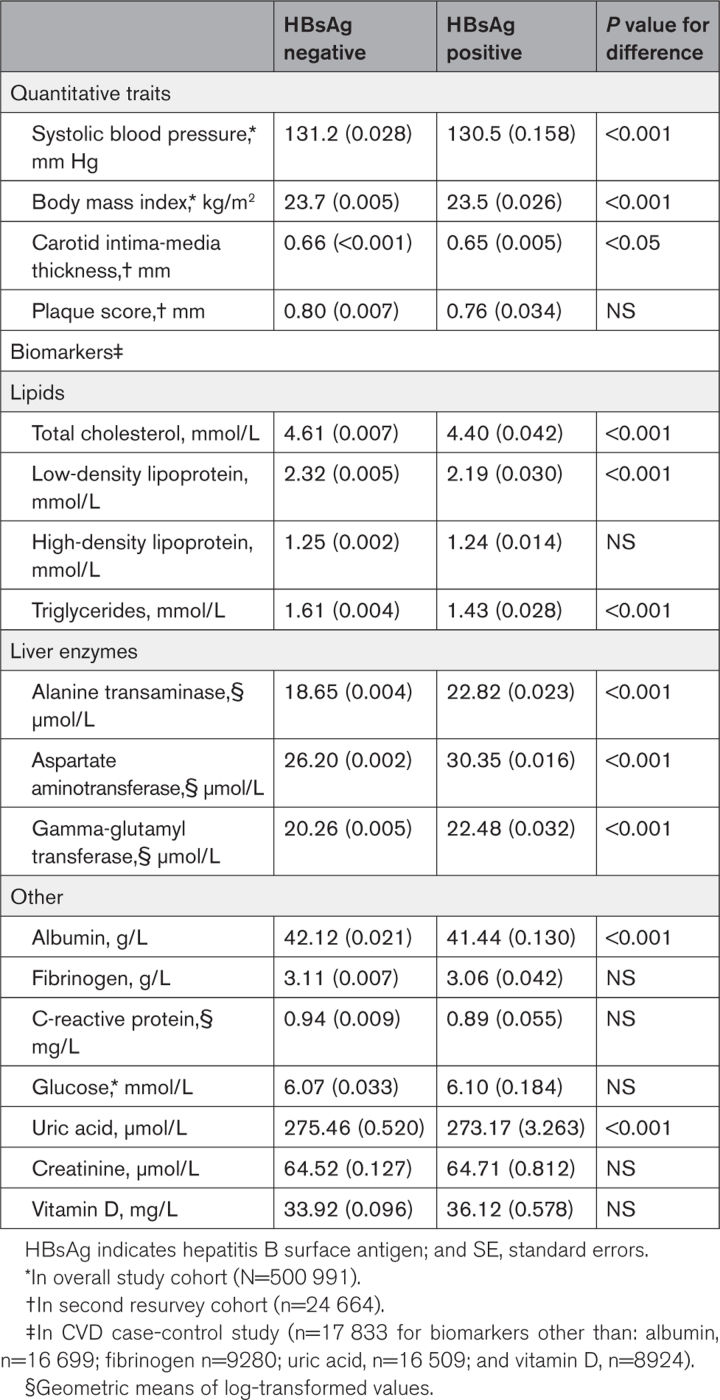
Adjusted Mean (SE) Values of Metabolic Traits and Blood Biochemistry by HBsAg Status

There was evidence of some mediation of the HBV-ICH association based on liver enzymes and albumin, with attenuation of the OR with the addition of liver enzymes and albumin to the model. After adding liver enzymes to the model, the OR was attenuated from 1.28 (1.01–1.62) to 1.22 (0.95–1.56; *P*<0.001), and further addition of albumin attenuated the association to 1.15 (0.90–1.48; *P*<0.001; Table S12). In stratified analyses by abnormal liver biomarkers, there was a tendency toward higher ORs for ICH associated with HBsAg positivity compared with HBsAg-negative participants, but these differences were not statistically significant (Figure S6). For lipids, there was no attenuation of the OR when they were added to the base model (Table S12).

## DISCUSSION

In this large prospective cohort study of 0.5 million Chinese men and women, we examined the associations of chronic HBV infection with risks of incident stroke and stroke types. Among 3% of the study population who were HBsAg-positive, the risk of ICH was significantly elevated compared with HBsAg-negative counterparts. There were no significant associations of HBsAg positivity with risks of IS and other stroke types. Chronic HBV infection was associated with elevated liver enzymes and lower lipid levels, and the HBV-ICH association may be mediated partially by abnormal liver function and, possibly to a smaller extent, by an altered lipid profile. While the population-attributable fraction was small, among HBsAg carriers, one-quarter of ICH cases could be attributed to infection, highlighting the need for consideration among people living with chronic HBV.

Few cohort studies have examined the associations of chronic HBV with the risk of stroke, with inconsistent findings. In a meta-analysis of 4 cohorts and 1 cross-sectional study conducted in East Asian^17,18^ and Western^11,19^ populations, chronic HBV was associated with reduced risk of total stroke (≈6000 stroke cases; RR, 0.78 [95% CI, 0.70–0.86]), with no information for stroke type. Across different cohorts included in the meta-analysis, stroke outcomes were heterogeneous, with 1 reporting only total stroke mortality^18^ and 2 not specifying stroke type.^11,19^ To our knowledge, only 2 cohort studies,^16,17^ included in this meta-analysis, reported the association of HBV with risk of stroke types, with 1^16^ reporting ICH and IS and 1 reporting only IS.^17^ In the former study, an 11-year occupational cohort study of 500 000 South Korean male public servants, there was a positive association of HBsAg positivity with risk of ICH (2523 events, HR, 1.33 [1.15–1.52]) and an inverse association with IS (4223 events, 0.79 [0.68–0.90]), with adjustment consistent with the present study. The HRs for both ICH and IS were more pronounced among participants with abnormal liver tests. The ratio of incident ICH to IS cases in this study was not consistent with that described previously.^31^ In the other 8-year retrospective cohort study^17^ of 110 000 Taiwanese adults in the National Health Insurance Research Database, chronic HBV was associated with a lower risk of IS (1490 cases; HR, 0.77 [95% CI, 0.66–0.89]), but ICH was not reported. This study used hospital diagnoses to ascertain HBsAg status and did not report the use of neuroimaging, and adjustment did not include important lifestyle factors (eg, smoking and alcohol intake).

The excess risk of ICH associated with HBsAg positivity in the present study was similar to the Korean study. However, we did not find an inverse association between HBsAg positivity and IS risk, including among adjudicated cases. Moreover, we found no clear association between chronic HBV and carotid atherosclerosis, consistent with a null association with IS.^22^ For carotid atherosclerosis, past evidence is divergent, where 2 studies^19,32^ in Europeans found no association with HBV, 1 study in Europeans reported an inverse association,^33^ and 1 Japanese study found a positive correlation.^34^ However, these studies were case-control or cross-sectional studies with low power (<50 HBsAg-positive participants), and an unclear exposure, with several combining chronic HBV and HCV infection.^19,32,33^ The present prospective study, with relatively low statin use, refutes any clear inverse associations of HBV infection with carotid atherosclerosis and risk of IS.

The mechanisms underlying the HBV-ICH association remain unclear and may be partially mediated by liver dysfunction, which can cause an anticoagulant state predisposing to bleeding. Although we did not have coagulation markers, we found that hypoalbuminemia may partially mediate the HBV-ICH association, which is a marker of the liver’s biosynthetic capacity. It is also possible that the HBV-ICH association may be linked to lipid metabolism, as there is evidence of impairment of host cell lipid metabolism via HBV antigens and HBV gene expression manipulating key transcription factors involved in lipid metabolism.^10^ A study on HCV and ICH has also shown that low lipid levels may contribute to vessel wall friability, and past studies have reported inverse associations of chronic HBV with cholesterol and triglyceride levels.^10,11^ Moreover, there is genetic and randomized trial evidence that lower levels of low-density lipoprotein-cholesterol increase the risk of ICH, with each 1 mmol/L lower low-density lipoprotein-cholesterol associated with 17% (95% CI, 3%–32%) higher risk of ICH.^25,35^

The strengths of this study include its prospective design, a large number of well-characterized (>90% diagnoses confirmed by neuroimaging) stroke cases (10× more IS and 4× more ICH cases than previous studies combined), and completeness of follow-up and ability to assess the consistency of the association for both fatal and nonfatal strokes. However, the study also had limitations. First, the HBsAg test has lower sensitivity than ELISA,^36^ which may lead to false negative results, and there were a modest number of participants with missing or unclear HBsAg data. Second, chronic HBV diagnosis was based on a single test, whereas 2 HBsAg-positive tests 6 months apart are the gold standard. Third, we lacked information to classify HBV disease severity, including HBV DNA levels, e-antigen status, serial liver enzyme markers, liver fibrosis measures, platelets, and coagulation status, preventing in-depth exploration of mechanisms underlying the HBV-ICH association. Fourth, biochemistry data were available only in a subset of participants, so the analyses would be underpowered. Fifth, we lacked information about HBV vaccination status or treatment during follow-up. However, this cohort is of the prevaccine era in China and is largely unvaccinated, and HBV treatment in China is low,^37^ so this is unlikely to materially affect the results. We also lacked data on other factors that may impact chronic hepatitis or stroke risk, including injecting drug use and sexual risk factors. Finally, this is an observational study, and thus, it is unable to investigate causality or eliminate unmeasured confounding.

## CONCLUSIONS

Overall, our findings provide robust new evidence supporting the link between chronic HBV infection and the risk of ICH in Chinese adults. Further research is needed to clarify the causal nature of the association and underlying biological mechanisms, including the potential role of host and viral genetics. Our findings may inform clinical decision-making about the potential risks and benefits of antithrombotic therapies among people with chronic HBV.

## ARTICLE INFORMATION

### Sources of Funding

The China Kadoorie Biobank baseline survey and the first resurvey were supported by the Kadoorie Charitable Foundation in Hong Kong. Long-term follow-up has been supported by Wellcome grants to Oxford University (212946/Z/18/Z, 202922/Z/16/Z, 104085/Z/14/Z, and 088158/Z/09/Z) and grants from the National Natural Science Foundation of China (82192900, 82192901, and 82192904) and the National Key Research and Development Program of China (2016YFC0900500). The UK Medical Research Council (MC_UU_00017/1, MC_UU_12026/2, and MC_U137686851), Cancer Research UK (C16077/A29186 and C500/A16896), and the British Heart Foundation (CH/1996001/9454) provided core funding to the Clinical Trial Service Unit and Epidemiological Studies Unit, Oxford University, for the project. Dr Hamilton is a recipient of an National Institute for Health and Care Research (NIHR) Biomedical Research Center Award supporting DPhil studies (NIHR-INF-1266). This research was funded in whole, or in part, by the Wellcome Trust (212946/Z/18/Z, 202922/Z/16/Z, 104085/Z/14/Z, and 088158/Z/09/Z).

### Disclosures

P.C. Matthews receives grants from GlaxoSmithKline, NIHR, and Wellcome Trust. The other authors report no conflicts.

### Supplemental Material

STROBE Checklist

Supplemental Methods

Tables S1–S12

Figures S1–S6

## Supplementary Material
