# Supplementary material for: Chronic Hepatitis B Virus Infection and Risk of Stroke Types: A Prospective Cohort Study of 500 000 Chinese Adults
Source: Stroke. 2023 Nov 9;54(12):3046–53. doi: 10.1161/STROKEAHA.123.043327 (PMC10664797; doi:10.1161/STROKEAHA.123.043327)
Supplement: Supplementary file 1 [file str-54-3046-s001.pdf]

SUPPLEMENTAL MATERIAL

Chronic hepatitis B virus infection and risk of stroke types: a prospective cohort study of 500,000 Chinese adults

Elizabeth M Hamilton[1], MD; Ling Yang[1,2,*], PhD; Neil Wright[1], PhD; Iain Turnbull[1], MRCP; Alexander J Mentzer[3], DPhil; Philippa C Matthews[4,5], DPhil; Yiping Chen[1], DPhil; Huaidong Du[1], PhD; Christiana Kartsonaki[1,2], DPhil; Yuanjie Pang[6,7], DPhil; Pei Pei[7], Bsc; Huizi Tian[8], MSc; Xiaoming Yang[1], PhD; Daniel Avery[1], MSc; Canqing Yu[6,7], PhD; Jun Lv[6,7], PhD; Robert Clarke[1], MD; Liming Li[6,7], MPH; Iona Y. Millwood[1,2,*], DPhil; Zhengming Chen[1,2,*], DPhil

1. Clinical Trial Service Unit & Epidemiological Studies Unit (CTSU), Nuffield Department of Population Health, University of Oxford, UK
2. Medical Research Council Population Health Research Unit (MRC PHRU), Nuffield Department of Population Health, University of Oxford, UK
3. The Wellcome Centre for Human Genetics, University of Oxford, UK
4. Division of Infection and Immunity, University College London, London, UK
5. The Francis Crick Institute, London, UK
6. Department of Epidemiology and Biostatistics, School of Public Health, Peking University Health Science Center, Beijing, China
7. Peking University Center for Public Health and Epidemic Preparedness and Response, Beijing, China
8. Non-Communicable Diseases Prevention and Control Department, Henan CDC, China

*Co-senior authors

Address for correspondence:

Dr Ling Yang
CTSU, Nuffield Department of Population Health
University of Oxford
Oxford OX3 7LF, UK
Email: ling.yang@ndph.ox.ac.uk

Table of Contents

Supplemental Strengthening the Reporting of Observational Studies in Epidemiology (STROBE) Checklist for cohort studies 3

Supplemental Methods 5

Table S1. Characteristics of participants with missing compared to non-missing data in Sichuan and Zhejiang, and unclear data in the whole cohort 6

Table S2. Test of proportionality for HBsAg-ICH cox proportional hazards model 1* 7

Table S3. Baseline characteristics of participants in second resurvey by HBsAg status 8

Table S4. Baseline characteristics of participants in CVD case-control study 9

Table S5. Cumulative incidence of stroke types in 5-year intervals by HBsAg status 10

Table S6. Risk of adjudicated IS and ICH by HBsAg status 11

Table S7. Risk of ICH by HBsAg status stratified by study site 12

Table S8. Risk of IS by HBsAg status stratified by study site 13

Table S9. Risk of stroke events by HBsAg status, excluding participants with a history of liver disease, cardiovascular disease or cancer at baseline 14

Table S10. Risk of stroke events by HBsAg status excluding first 2 years of follow-up 15

Table S11. Risk of first stroke event of any type by HBsAg status 16

Table S12. Change in HBV-ICH model fit with addition of lipids and LFTs among participants with biochemistry data (N= 17,833 participants)* 17

Figure S1. Flow diagram for analytical sample 18

Figure S2. Schoenfeld residual tests for HBsAg and ICH Cox proportional hazards model 19

Figure S3. Prevalence of Hepatitis B surface antigen by age, sex and study area 20

Figure S5. Adjusted HRs for a) ICH and b) IS associated with by HBsAg positivity in selected population subgroups 22

Figure S6. Risk of ICH by HBsAg status stratified by abnormal LFTs, albumin and liver abnormality score among participants with biochemistry data 23

Supplemental Strengthening the Reporting of Observational Studies in Epidemiology (STROBE) Checklist for cohort studies

	Item No	Recommendation	Page,
**Title and abstract**	1	(*a*) Indicate the study's design with a commonly used term in the title or the abstract	Title and abstract
		(*b*) Provide in the abstract an informative and balanced summary of what was done and what was found	Title and abstract
**Introduction**			
Background/rationale	2	Explain the scientific background and rationale for the investigation being reported	p.1
Objectives	3	State specific objectives, including any prespecified hypotheses	p.2
**Methods**			
Study design	4	Present key elements of study design early in the paper	p.2
Setting	5	Describe the setting, locations, and relevant dates, including periods of recruitment, exposure, follow-up, and data collection	p.2-3
Participants	6	(*a*) Give the eligibility criteria, and the sources and methods of selection of participants. Describe methods of follow-up	p.2
		(*b*) For matched studies, give matching criteria and number of exposed and unexposed	NA
Variables	7	Clearly define all outcomes, exposures, predictors, potential confounders, and effect modifiers. Give diagnostic criteria, if applicable	p.2-3
Data sources/ measurement	8*	For each variable of interest, give sources of data and details of methods of assessment (measurement). Describe comparability of assessment methods if there is more than one group	p.2-3
Bias	9	Describe any efforts to address potential sources of bias	p.3
Study size	10	Explain how the study size was arrived at	-
Quantitative variables	11	Explain how quantitative variables were handled in the analyses. If applicable, describe which groupings were chosen and why	p.3
Statistical methods	12	(*a*) Describe all statistical methods, including those used to control for confounding	p.3
		(*b*) Describe any methods used to examine subgroups and interactions	p.3
		(*c*) Explain how missing data were addressed	p.3
		(*d*) If applicable, explain how loss to follow-up was addressed	-
		(*e*) Describe any sensitivity analyses	p.3.
**Results**			
Participants	13*	(a) Report numbers of individuals at each stage of study—eg numbers potentially eligible, examined for eligibility, confirmed eligible, included in the study, completing follow-up, and analysed	p.3
		(b) Give reasons for non-participation at each stage	As above
		(c) Consider use of a flow diagram	As above
Descriptive data	14*	(a) Give characteristics of study participants (eg demographic, clinical, social) and information on exposures and potential confounders	p.3-4
		(b) Indicate number of participants with missing data for each variable of interest	p.3
		(c) Summarise follow-up time (eg, average and total amount)	p.3-4
Outcome data	15*	Report numbers of outcome events or summary measures over time	p.3-4
Main results	16	(*a*) Give unadjusted estimates and, if applicable, confounder-adjusted estimates and their precision (eg, 95% confidence interval). Make clear which confounders were adjusted for and why they were included	p.4
		(*b*) Report category boundaries when continuous variables were categorized	p.3
		(*c*) If relevant, consider translating estimates of relative risk into absolute risk for a meaningful time period	p.4
Other analyses	17	Report other analyses done—eg analyses of subgroups and interactions, and sensitivity analyses	p.4-5

**Discussion**			
Key results	18	Summarise key results with reference to study objectives	p.5-6
Limitations	19	Discuss limitations of the study, taking into account sources of potential bias or imprecision. Discuss both direction and magnitude of any potential bias	p.6-7
Interpretation	20	Give a cautious overall interpretation of results considering objectives, limitations, multiplicity of analyses, results from similar studies, and other relevant evidence	p.7
Generalisability	21	Discuss the generalisability (external validity) of the study results	p.7
**Other information**			
Funding	22	Give the source of funding and the role of the funders for the present study and, if applicable, for the original study on which the present article is based	p.7

*Give information separately for exposed and unexposed groups.

Available: https://www.strobe-statement.org/checklists/

Supplemental Methods

Inverse probability weighting

As described in past CKB work,[30] inverse probability of sampling weights (i.e. inclusion in the cardiovascular nested case-control study) were developed to ensure analysis accounted for inclusions/exclusion criteria and sampling scheme for the nested case-control study. Cases and controls were assigned different weights to reflect the different proportions of cases and controls from eligible participants in the entire CKB cohort. The weights were calculated separately for controls and cases as the number of eligible participants divided by the number selected in the nested case-control study. The weights were 72.35 for controls, 5.87 for IS cases and 1.62 for ICH cases.

Follow-up of long-term outcomes

Follow-up of long-term outcomes in CKB have been described in detail elsewhere.[20] The ten study sites are part of China's Disease Surveillance Points (DSP) system, which provides mortality statistics for the whole country. Vital status is monitored via official residential records and death certificates, which are reported to the regional coordinating centre at each study site, a team of people with relevant medical qualification and fieldwork experience. Where cause of death was unclear, more thorough review of hospital records or verbal autopsy was performed using a validated instrument. Non-fatal outcomes were identified by linkage to hospital records and disease registries. Using participant's unique national identification codes, participants were linked to health insurance claims databases, which has >98% coverage across study areas. Additionally, several major diseases included stroke, IHD, cancer and diabetes have disease registries that are available in 8 of 10 study areas . All events are coded using the International Classification of Disease 10th edition (ICD-10) by trained staff who are blinded to baseline information. Active confirmation of vital status and non-fatal outcomes through health insurance and administrative records was also carried out annually to reduce under-reporting and loss-to-follow up.

Carotid artery measurements

As described in past CKB work[21,22] details of carotid artery measurement protocols are as follows: Automated B-mode ultrasound screening of the extra-cranial carotid arteries (using a Panasonic Cardio-Health Station implementing edge-detection software) was undertaken following a standard protocol consistent with the Mannheim consensus, and involved scanning both carotid arteries with automated measurements of carotid intima-media thickness (cIMT) and semi-automated recording of plaques. cIMT was measured in the distal 1cm of the common carotid artery (CCA) just before the bifurcation at four predefined angles (two on each side), using an inbuilt electronic transducer position guidance, including the right CCA at 150º and 120º and the left CCA at 210º and 240º. Mean cIMT was estimated as the mean of four measurements per person. The number of carotid plaques (defined as focal thickenings of intima-media >1.5 mm) and the thickness of the largest plaque within four segments of the carotid arteries were recorded. Carotid plaque burden was derived by first standardising the plaque number and maximum thickness (i.e., dividing each by its standard deviation [SD]) and estimating the average, then multiplying the average value by the SD of the maximum plaque thickness to provide a plaque burden recorded in millimetre units (i.e. interpretable as an enhanced estimate of the maximum plaque thickness).

Table S1. Characteristics of participants with missing compared to non-missing data in Sichuan and Zhejiang, and unclear data in the whole cohort

	Sichuan			Zhejiang			Whole cohort		
	**Not missing** n=52,138	**Missing** n=3,548	**p-value***	**Not missing** n=53,840	**Missing** n=3,864	**p-value***	**Not unclear** n=500,991	**Unclear** n=3,539	**p-value***
**Age(years), mean (SD)**	51.6 (10.5)	50.4 (10.7)	<0.001	53.0 (9.92)	50.6 (9.80)	<0.001	52.1 (10.7)	51.0 (10.4)	<0.001
**Men, %**	38.0	42.6	<0.001	41.7	41.0	NS	41.0	41.1	NS
**No formal education, %**	15.4	16.5	NS	42.8	61.5	<0.001	18.2	25.4	<0.001
**Ever-regular smoker†, %**	38.1	42.3	<0.001	35.1	35.8	NS	32.3	32.2	NS
**Current alcohol intake‡, %**	32.7	41.5	<0.001	22.0	23.7	<0.05	22.2	17.9	<0.001
**Dietary factors, regular intake§, %**									
Fruit	20.9	31.4	<0.001	16.6	29.2	<0.001	28.1	31.8	<0.001
Meat	42.0	50.4	<0.001	48.8	42.1	<0.001	47.2	46.3	NS
Dairy	3.74	4.45	<0.05	1.17	0.91	NS	12	12.9	NS
**Physical activity (MET-hours/day), mean (SD)**	22.1 (11.6)	22.3 (13.7)	NS	30.0 (15.2)	33.3 (16.9)	<0.001	21.0 (13.8)	23.8 (14.6)	<0.001
**SBP (mmHg), mean (SD)**	129 (18.8)	126 (21.2)	<0.001	136 (21.4)	128 (19.9)	<0.001	131 (21.3)	130 (20.6)	<0.01
**BMI (kg/m^2), mean (SD)**	23.3 (3.20)	22.8 (3.16)	<0.001	23.0 (3.16)	22.8 (3.12)	NS	23.7 (3.38)	23.5 (3.29)	<0.01
**Prior disease history, %**									
Cancer	0.22	0.28	NS	0.27	0.49	<0.05	0.5	0.59	NS
Chronic liver disease	1.58	3.38	<0.001	2.06	2.10	NS	1.17	3.11	<0.001
Diabetes	1.72	1.04	<0.01	2.54	2.07	NS	3.17	2.46	<0.05

*****p-value obtained from t-test for continuous variables or chi-squared test for categorical variables comparing HBsAg negative and positive participants, †ever-regular smoker includes ex and current smokers, never regular includes never and occasional smoking, ‡ no current alcohol intake includes participants reporting monthly, occasional or ex–regular alcohol intake; current includes weekly and reduced intake, § regular intake includes participants report intake 4 or more times per week. Abbreviations: HBsAg, hepatitis B surface antigen; MET, metabolic equivalent of task; SBP, systolic blood pressure.

Table S2. Test of proportionality for HBsAg-ICH cox proportional hazards model 1*

Variable	Chi-squared	Degree of freedom	p-value
**HBsAg**	0.4	1	NS
**Education**	5.3	2	NS
**Income**	0.8	2	NS
**Global test**	6.7	5	NS

*stratified for age (5-year groups), sex, study-site (10 sites) and adjusted for education (three levels) and income (3 levels). Abbreviations: HBsAg, hepatitis B surface antigen

Table S3. Baseline characteristics of participants in second resurvey by HBsAg status

	Overall (N=24,774)	HBsAg Negative (n=24,031)	HBsAg positive (n=743)	p-value*
**Age (years),** mean (SD)	51.6 (10.1)	51.7 (10.2)	49.3 (9.5)	<0.05
**Men,** %	38.4	38.3	39.2	NS
**Rural residence, %**	56.5	56.9	44.5	<0.001
**No formal education,** %	18.9	18.8	17.8	NS
**Ever-regular smoker†, %**				
Men	74.3	74.0	67.9	<0.01
Women	2.7	2.6	2.8	NS
**Current alcohol intake‡,** %				
Men	39.4	39.2	30.6	<0.05
Women	2.5	2.5	2.3	NS
**Dietary factors, regular intake§,** %				
Fruit	27.1	27.2	24.9	NS
Dairy	10.7	10.6	12.7	NS
Meat	49.5	49.6	45.4	NS
**BMI, kg/m^2,** mean (SD)	23.8 (3.7)	23.8 (3.7)	21.9 (3.3)	<0.05
**SBP, mmHg,** mean (SD)	131.1 (20.8)	131.1 (20.8)	120.7 (20.1)	<0.05
**Physical activity** (MET-hours/day), mean (SD)	21.4 (14.0)	21.4 (14.0)	20.0 (14.8)	<0.05
**Prior disease history,** %				
Chronic liver disease	1.0	0.6	10.8	NS
Diabetes	2.6	2.7	2.4	NS

***p-value obtained from t-test for continuous variables or chi-squared test for categorical variables comparing HBsAg negative and positive participants, †ever-regular smoker includes ex and current smokers, never regular includes never and occasional smoking, ‡ no current alcohol intake includes participants reporting monthly, occasional or ex–regular alcohol intake; current includes weekly and reduced intake, § regular intake includes participants report intake 4 or more times per week. Abbreviations: HBsAg, hepatitis B surface antigen; MET, metabolic equivalent of task; SBP, systolic blood pressure.

Table S4. Baseline characteristics of participants in CVD case-control study

	Overall (N=17,833)	HBsAg negative (n=17,367)	HBsAg positive (n=466)	p-value*
**Age (years),** mean (SD)	56.9 (10.2)	57.0 (10.2)	53.9 (10.5)	<0.05
**Men,** %	49.1	49.2	50.5	NS
**Rural residence, %**	69.8	69.9	64.2	NS
**No formal education,** %	23.7	23.7	23.6	NS
**Ever-regular smoker†, %**				
Men	77.5	75.0	76.0	<0.01
Women	4.6	4.6	5.4	NS
**Current alcohol intake ‡,** %				
Men	35.5	35.6	32.5	<0.05
Women	2.5	2.5	2.8	NS
**Dietary factors, regular intake§,** %				
Fruit	19.7	19.8	15.1	NS
Dairy	9.9	9.8	11.2	NS
Meat	37.6	37.7	32.5	NS
**BMI,** kg/m^2	23.6 (3.3)	23.6 (3.3)	21.5 (3.4)	<0.05
**SBP,** mmHg	142.1 (24.6)	142.1 (24.6)	130.3 (22.4)	<0.05
**Physical activity** (MET–hours/day), mean (SD)	18.3 (11.3)	18.4 (11.4)	15.5 (9.3)	NS
**Prior disease history,** %				
Chronic liver disease	1.1	0.8	9.3	<0.01
Diabetes	4.6	4.7	2.2	NS

*****p-value obtained from t-test for continuous variables or chi-squared test for categorical variables comparing HBsAg negative and positive participants, †ever-regular smoker includes ex and current smokers, never regular includes never and occasional smoking, ‡ no current alcohol intake includes participants reporting monthly, occasional or ex–regular alcohol intake; current includes weekly and reduced intake, § regular intake includes participants report intake 4 or more times per week. Abbreviations: HBsAg, hepatitis B surface antigen; MET, metabolic equivalent of task; SBP, systolic blood pressure.

Table S5. Cumulative incidence of stroke types in 5-year intervals by HBsAg status

Stroke type	HBsAg status	Cumulative incidence (%, 95 CI %)						Gray's test*
		50 years	55 years	60 years	65 years	70 years	75 years	
**ICH**	-	0.17 (0.16–0.19)	0.38 (0.36–0.40)	0.78 (0.75–0.81)	1.4 (1.4–1.5)	2.5 (2.4–2.6)	4.4 (4.3–4.5)	<0.01
	+	0.25 (0.18–0.34)	0.60 (0.47–0.74)	1.1 (0.92–1.3)	2.0 (1.7–2.3)	3.2 (2.8–3.7)	4.8 (4.1–5.4)	
**IS**	-	0.56 (0.54–0.58)	1.6 (1.5–1.6)	3.6 (3.5–3.6)	6.8 (6.7–6.9)	12 (11–12)	19 (19–19)	<0.01
	+	0.52 (0.41–0.64)	1.6 (1.3–1.8)	3.6 (3.3–4.0)	7.1 (6.6–7.7)	12 (11–13)	18 (17–19)	
**SAH**	-	0.02 (0.02–0.03)	0.05 (0.04–0.06)	0.10 (0.09–0.11)	0.16 (0.15–0.17)	0.25 (0.23–0.27)	0.38 (0.35–0.41)	NS
	+	0.03 (0.01–0.07)	0.06 (0.03–0.12)	0.09 (0.05–0.17)	0.12 (0.07–0.21)	0.26 (0.15–0.43)	0.30 (0.17–0.50)	
**Other**	-	0.03 (0.03–0.04)	0.09 (0.08–0.10)	0.21 (0.19–0.22)	0.38 (0.36–0.41)	0.64 (0.61–0.68)	1.1 (1.1–1.2)	NS
	+	0.03 (0.01–0.07)	0.09 (0.05–0.16)	0.21 (0.13–0.31)	0.37 (0.26–0.52)	0.82 (0.61–1.10)	1.1 (0.80–1.4)	
**Total**	-	0.74 (0.71–0.76)	2.0 (1.9–2.0)	4.3 (4.2–4.3)	8.0 (7.9–8.1)	14 (13–14)	22 (22–23)	NS
	+	0.76 (0.63–0.91)	2.1 (1.9–2.4)	4.7 (4.3–5.1)	8.8 (8.2–9.4)	15 (14–15)	22 (20–23)	

*Gray's test comparing cumulative incidence by HBsAg status using a modified chi-squared test.
Abbreviations: HBsAg, hepatitis B surface antigen; ICH, intracerebral hemorrhage; IS, ischemic stroke; SAH, subarachnoid hemorrhage.

Table S6. Risk of adjudicated IS and ICH by HBsAg status

Endpoint	Number of events		HR (95% CI)	p-value
	HBsAg Negative	HBsAg Positive		
**ICH**	3,818	123	1.28 (1.07–1.54)	<0.01
**IS**	21,765	507	0.95 (0.87–1.04)	NS

Abbreviations: HBsAg, hepatitis B surface antigen; HR, hazard ratio; ICH, intracerebral hemorrhage; IS, ischemic stroke.

Table S7. Risk of ICH by HBsAg status stratified by study site

Study site	HBsAg negative	HBsAg positive	HR (95% CI)*	p-value
Sichuan (Rural)	1126	26	1.86 (1.26–2.75)	<0.01
Suzhou (Urban)	540	31	1.69 (1.18–2.43)	<0.01
Henan (Rural)	1588	71	1.46 (1.15–1.86)	<0.01
Haikou (Urban)	393	21	1.31 (0.84–2.04)	NS
Gansu (Rural)	2054	37	1.29 (0.93–1.79)	NS
Harbin (Urban)	1127	25	1.26 (0.85–1.88)	NS
Liuzhou (Urban)	770	28	1.11 (0.76–1.61)	NS
Hunan (Rural)	2370	59	1.10 (0.85–1.43)	NS
Zhejiang (Rural)	749	22	1.08 (0.70–1.64)	NS
Qingdao (Urban)	275	6	0.91 (0.41–2.06)	NS

Test for heterogeneity across study sites: χ^2=9.9, df=9, p=NS. *Stratified by age (5-year categories) and sex, adjusted for education, smoking, alcohol, physical activity, regular fruit, meat or dairy intake, BMI, systolic blood pressure, prevalent diabetes and cancer. Abbreviations: ICH, intracerebral hemorrhage; HR, hazard ratio.

Table S8. Risk of IS by HBsAg status stratified by study site

Study site	HBsAg negative	HBsAg positive	HR (95% CI)*	p-value
Suzhou (Urban)	2340	86	1.18 (0.95–1.47)	NS
Haikou (Urban)	4056	181	1.08 (0.93–1.25)	NS
Liuzhou (Urban)	4389	150	1.07 (0.91–1.26)	NS
Sichuan (Rural)	2648	34	0.99 (0.71–1.39)	NS
Qingdao (Urban)	1742	36	0.96 (0.69–1.33)	NS
Hunan (Rural)	4821	101	0.94 (0.77–1.15)	NS
Harbin (Urban)	14045	222	0.93 (0.82–1.07)	NS
Zhejiang (Rural)	2269	57	0.91 (0.70–1.18)	NS
Gansu (Rural)	3823	54	0.89 (0.68–1.16)	NS
Henan (Rural)	8684	233	0.88 (0.77–1.00)	NS

Test for heterogeneity across study sites: χ^2=9.8, df=9, p=NS. *Stratified by age (5-year categories) and sex, adjusted for education, smoking, alcohol, physical activity, regular fruit, meat or dairy intake, BMI, systolic blood pressure, prevalent diabetes and cancer. Abbreviations: IS, ischemic stroke; HR, hazard ratio.

Table S9. Risk of stroke events by HBsAg status, excluding participants with a history of liver disease, cardiovascular disease or cancer at baseline

Endpoint	Number of events		HR (95% CI)*	p-value
	HBsAg negative	HBsAg positive		
ICH	9,351	256	1.27 (1.12–1.44)	<0.001
IS	40,554	874	0.94 (0.88–1.00)	NS
SAH	862	16	0.77 (0.47–1.26)	NS
Other stroke	2,383	48	0.99 (0.75–1.32)	NS
Total stroke	48,314	1,097	0.99 (0.93–1.05)	NS

*Stratified by age (5-year categories), sex, study site, adjusted for education, adjusted for smoking, alcohol, physical activity, regular fruit, meat or dairy intake, BMI, systolic blood pressure, prevalent diabetes. Abbreviations: HBsAg, hepatitis B surface antigen; ICH, intracerebral hemorrhage; IS, ischemic stroke; SAH, subarachnoid hemorrhage.

Table S10. Risk of stroke events by HBsAg status excluding first 2 years of follow-up

Endpoint	Number of events		HR (95% CI)*	p-value
	HBsAg negative	HBsAg positive		
ICH	9,621	280	1.27 (1.12–1.43)	<0.001
IS	44,123	1,048	0.97 (0.92–1.04)	NS
SAH	858	21	0.95 (0.62–1.47)	NS
Other stroke	2,762	66	1.12 (0.88–1.43)	NS
Total stroke	51,584	1,275	1.02 (0.96–1.08)	NS

*Stratified by age (5-year categories), sex, study site, adjusted for education, adjusted for smoking, alcohol, physical activity, regular fruit, meat or dairy intake, BMI, systolic blood pressure, prevalent diabetes. Abbreviations: HBsAg, hepatitis B surface antigen; ICH, intracerebral hemorrhage; IS, ischemic stroke; SAH, subarachnoid hemorrhage.

Table S11. Risk of first stroke event of any type by HBsAg status

Endpoint	Number of events		HR (95% CI)*	p-value
	HBsAg negative	HBsAg positive		
ICH	5,094	161	1.22 (1.05-1.43)	<0.01
IS	44,555	1,057	0.96 (0.91-1.03)	NS
SAH	581	12	0.75 (0.42-1.32)	NS
Other stroke	814	22	1.06 (0.69-1.61)	NS

*Stratified by age (5-year categories), sex, study site, adjusted for education, adjusted for smoking, alcohol, physical activity, regular fruit, meat or dairy intake, BMI, systolic blood pressure, prevalent diabetes. Abbreviations: HBsAg, hepatitis B surface antigen; ICH, intracerebral hemorrhage; IS, ischemic stroke; SAH, subarachnoid hemorrhage.

Table S12. Change in HBV-ICH model fit with addition of lipids and LFTs among participants with biochemistry data (N= 17,833 participants)*

Additional variable added to model	OR (95% CI)	Change in model fit†		
		χ^2	Degrees of freedom	P-value
**Base model‡**	1.28 (1.01–1.62)			
**Base model + CLD**	1.26 (0.99–1.60)	0.6	1	NS
**Base model + lipids**				
LDL (mmol/L)	1.28 (1.00–1.63)	0.1	1	NS
HDL (mmol/L)	1.27 (1.00–1.62)	1.8	1	NS
TG (mmol/L)	1.28 (1.00–1.63)	0.5	1	NS
LDL, HLD and TG (mmol/L)	1.29 (1.01–1.64)	6.9	3	NS
**Base model + liver biomarkers**				
ALT (μmol/L)	1.23 (0.97–1.58)	6.4	1	0.01
AST (μmol/L)	1.20 (0.94–1.53)	24.6	1	<0.001
GGT (μmol/L)	1.23 (0.97–1.58)	58.3	1	<0.001
ALT, AST, GGT (μmol/L)	1.22 (0.95–1.56)	61.2	3	<0.001
Albumin (g/L)	1.20 (0.94–1.54)	34.7	1	<0.001
ALT, AST, GGT (μmol/L), Albumin (g/L)	1.15 (0.90–1.48)	95.4	4	<0.001
**Addition of lipids to model containing LFTs and albumin**				
Base model + LFTs + albumin + lipids	1.16 (0.90–1.49)	3.82	3	NS

*Among 17,833 participants, where 466 are HBsAg positive and 17,367 are HBsAg negative. †Change in model fit obtained using likelihood ratio test comparing base model to model with additional variable. ‡Base model is adjusted for age (5-year categories), sex, region, education, alcohol and smoking. Abbreviations: HBV, hepatitis B virus; ICH, intracerebral hemorrhage; OR, odds ratio; CLD, chronic liver disease; LDL, low-density lipoprotein; HDL, high-density lipoprotein; TG, triglycerides; LFTs, liver function tests; ALT, alanine transaminase; AST, aspartate aminotransferase; GGT, Gamma-glutamyl transferase

Figure S1. Flow diagram for analytical sample

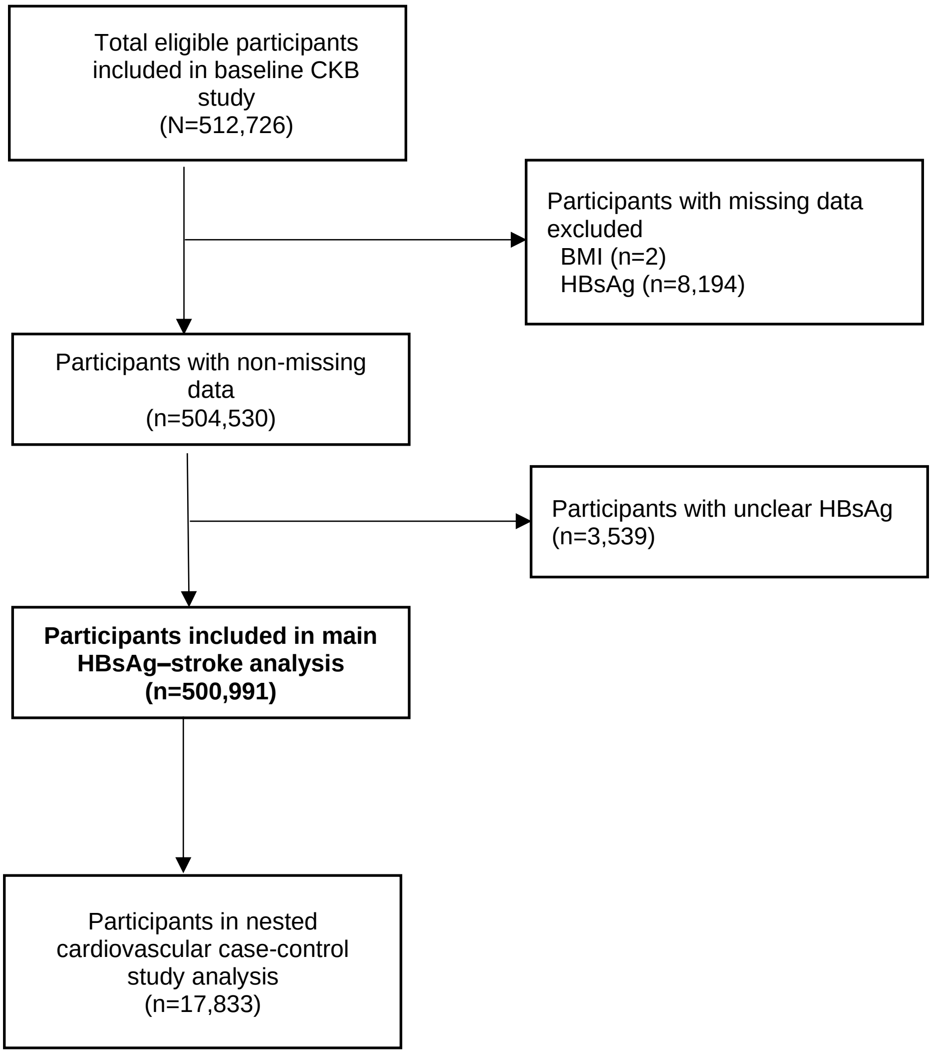

Abbreviations: BMI, body mass index; HBsAg, hepatitis B surface antigen

Figure S2. Schoenfeld residual tests for HBsAg and ICH Cox proportional hazards model

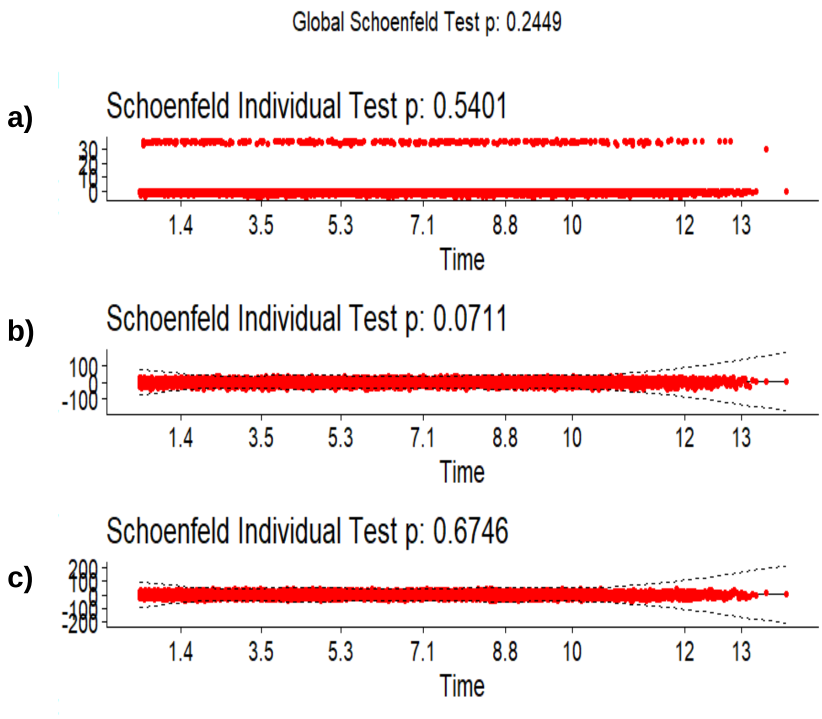

Figure shows residuals for HBsAg-ICH cox proportional hazard model 1, a) Hepatitis B surface antigen; b), education; c); income. Abbreviations: HBsAg, hepatitis B surface antigen; ICH, intracerebral hemorrhage

Figure S3. Prevalence of Hepatitis B surface antigen by age, sex and study area

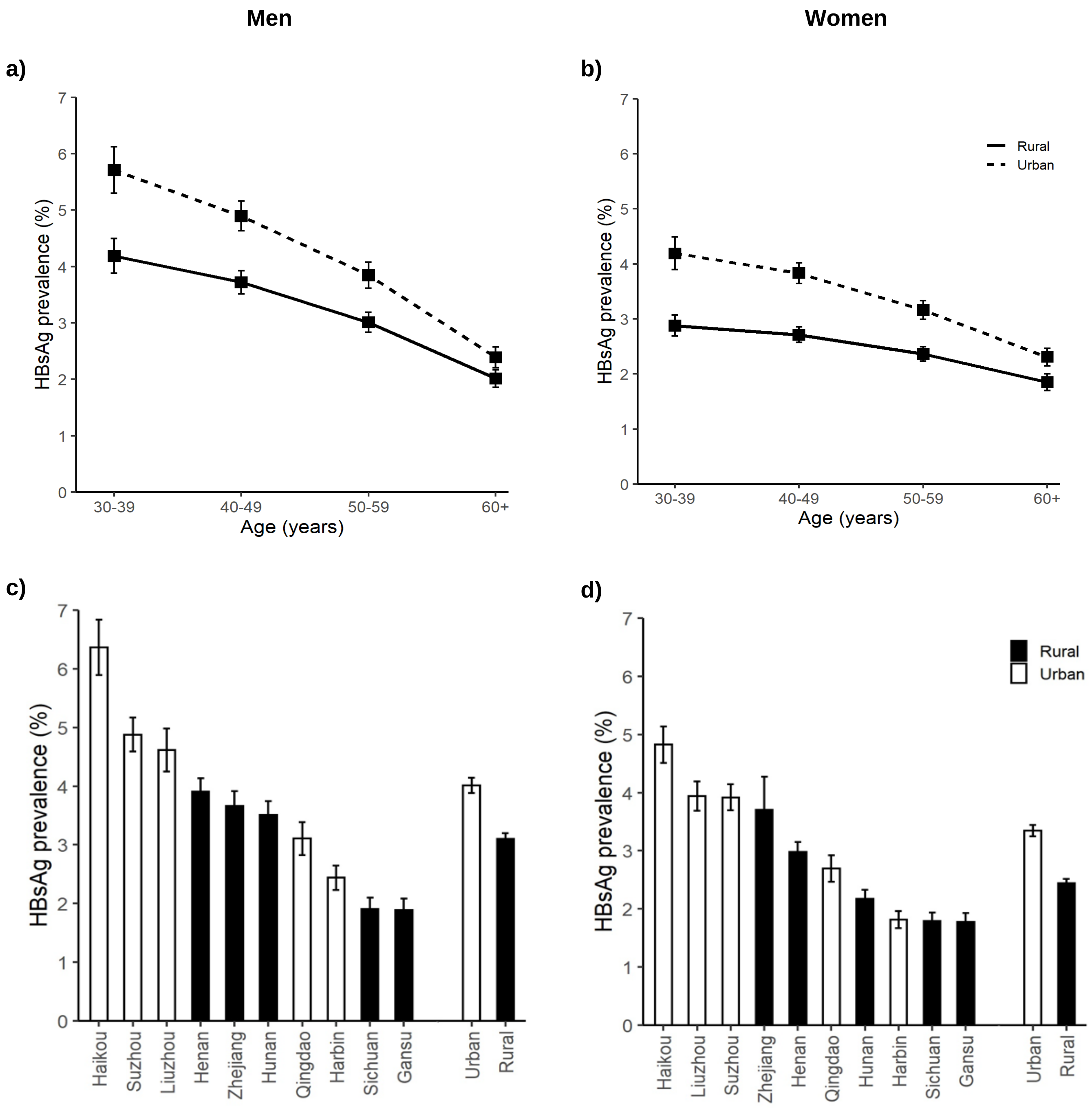

Hepatitis B surface antigen (HBsAg) prevalences displayed (95% CI) are standardized by age and study site among men and women separately, stratified by urban or rural location. a) HBsAg prevalence in men by age category, b) HBsAg prevalence in women by age category, c) HBsAg prevalence in men by study site, d) HBsAg prevalence in women by study site**.** Abbreviations: HBsAg, hepatitis B surface antigen.

Figure S4. Cumulative incidence of a) ICH, b) IS, c) SAH, d) other stroke and e) total stroke by HBsAg status

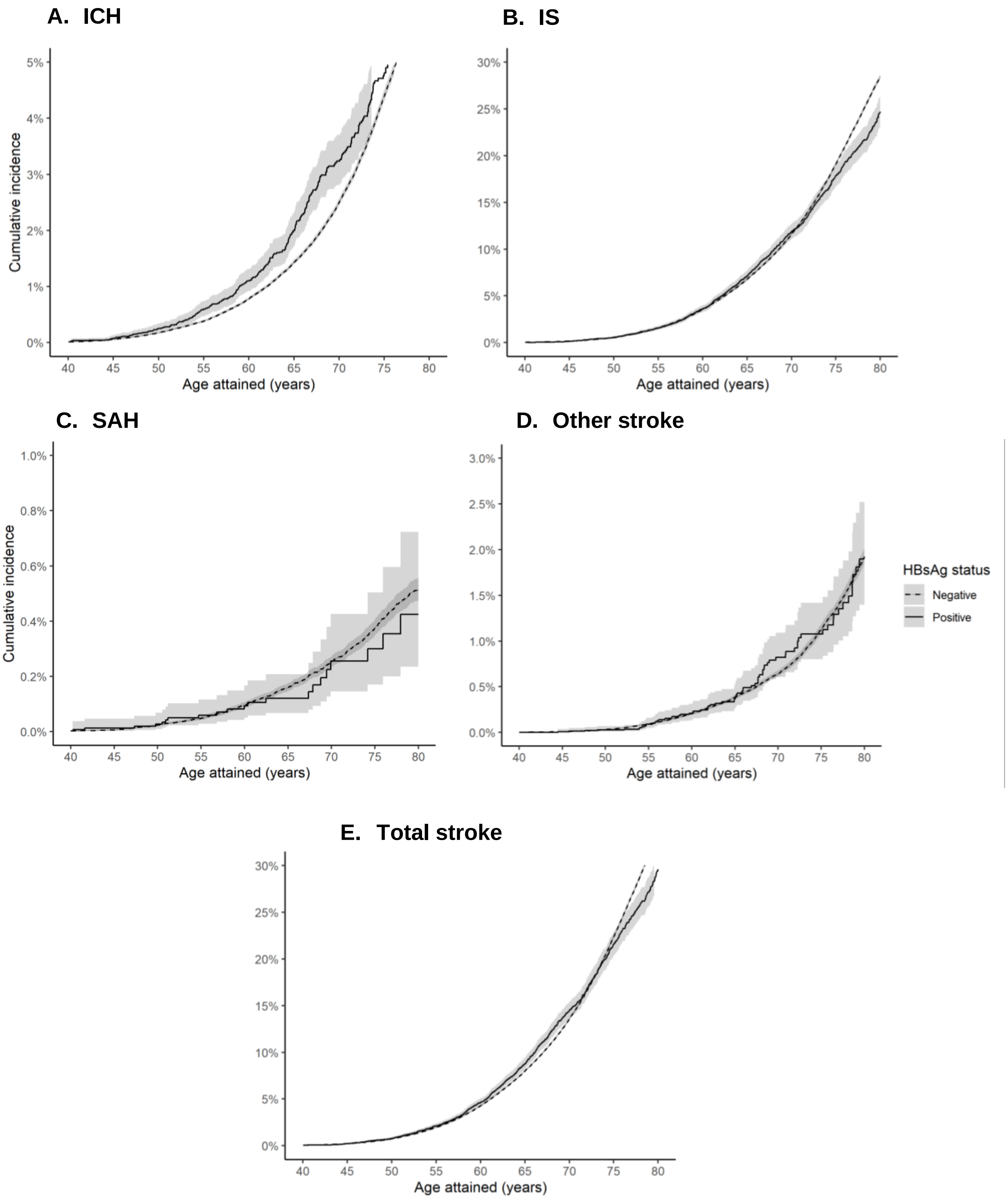

Abbreviations: HBsAg, hepatitis B surface antigen; ICH, intracerebral hemorrhage; IS, ischemic stroke; SAH, subarachnoid hemorrhage.

Figure S5. Adjusted HRs for a) ICH and b) IS associated with by HBsAg positivity in selected population subgroups

a) ICH **b) IS**

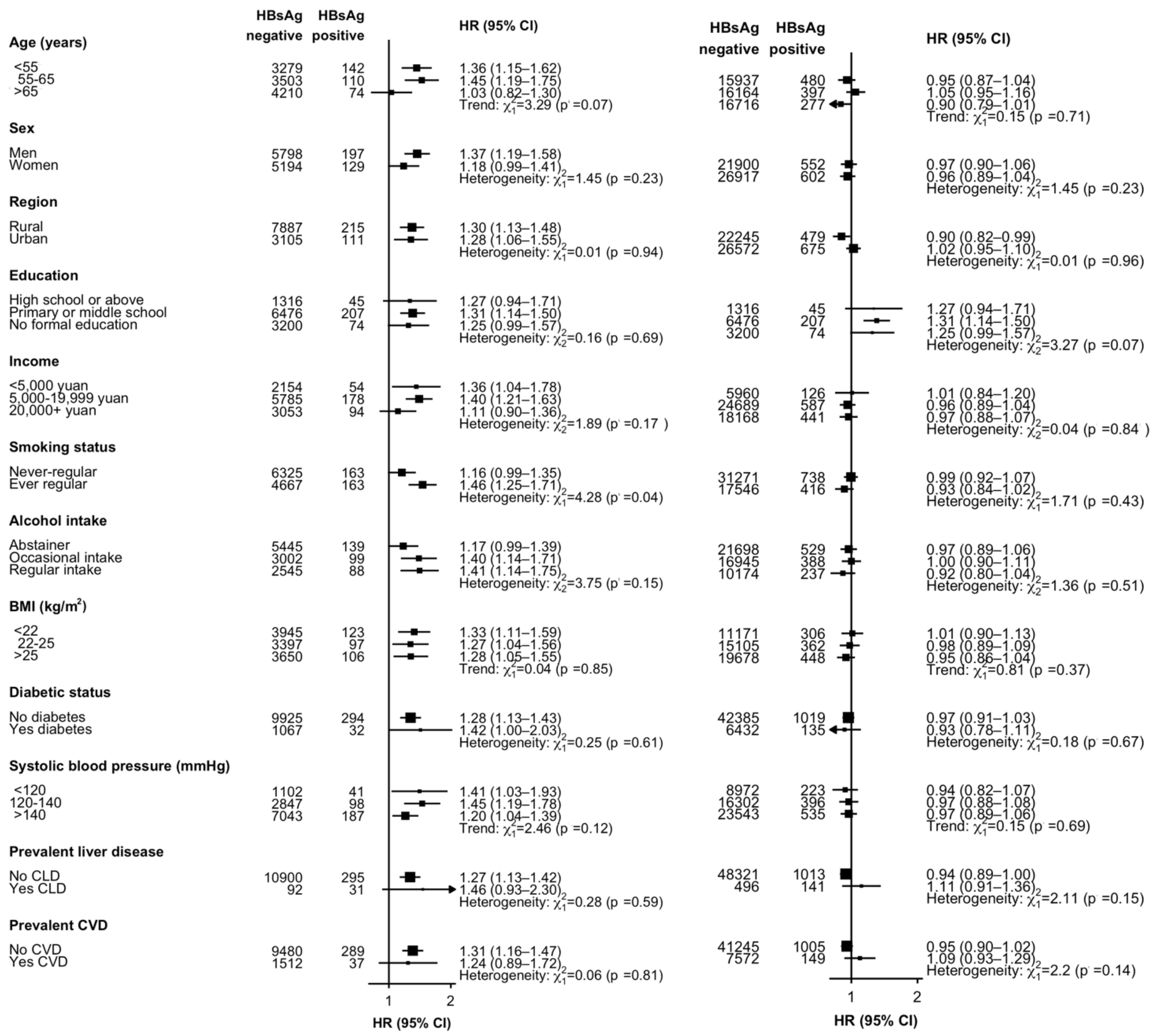

Prevalent liver disease includes chronic hepatitis, cirrhosis or liver cancer diagnosed by a doctor; prevalent CVD includes coronary artery disease, stroke, transient ischemic attack or rheumatic heart disease diagnosed by a doctor. Models were stratified for age (5-year categories), sex, study site, and adjusted for education, income, smoking, alcohol, physical activity, regular fruit, meat or dairy intake, BMI, systolic blood pressure, prevalent diabetes where possible. Abbreviations: ICH: intracerebral hemorrhage; IS: ischemic stroke; HBsAg, hepatitis B surface antigen; BMI, body mass index; and CVD, cardiovascular disease.

Figure S6. Risk of ICH by HBsAg status stratified by abnormal LFTs, albumin and liver abnormality score among participants with biochemistry data

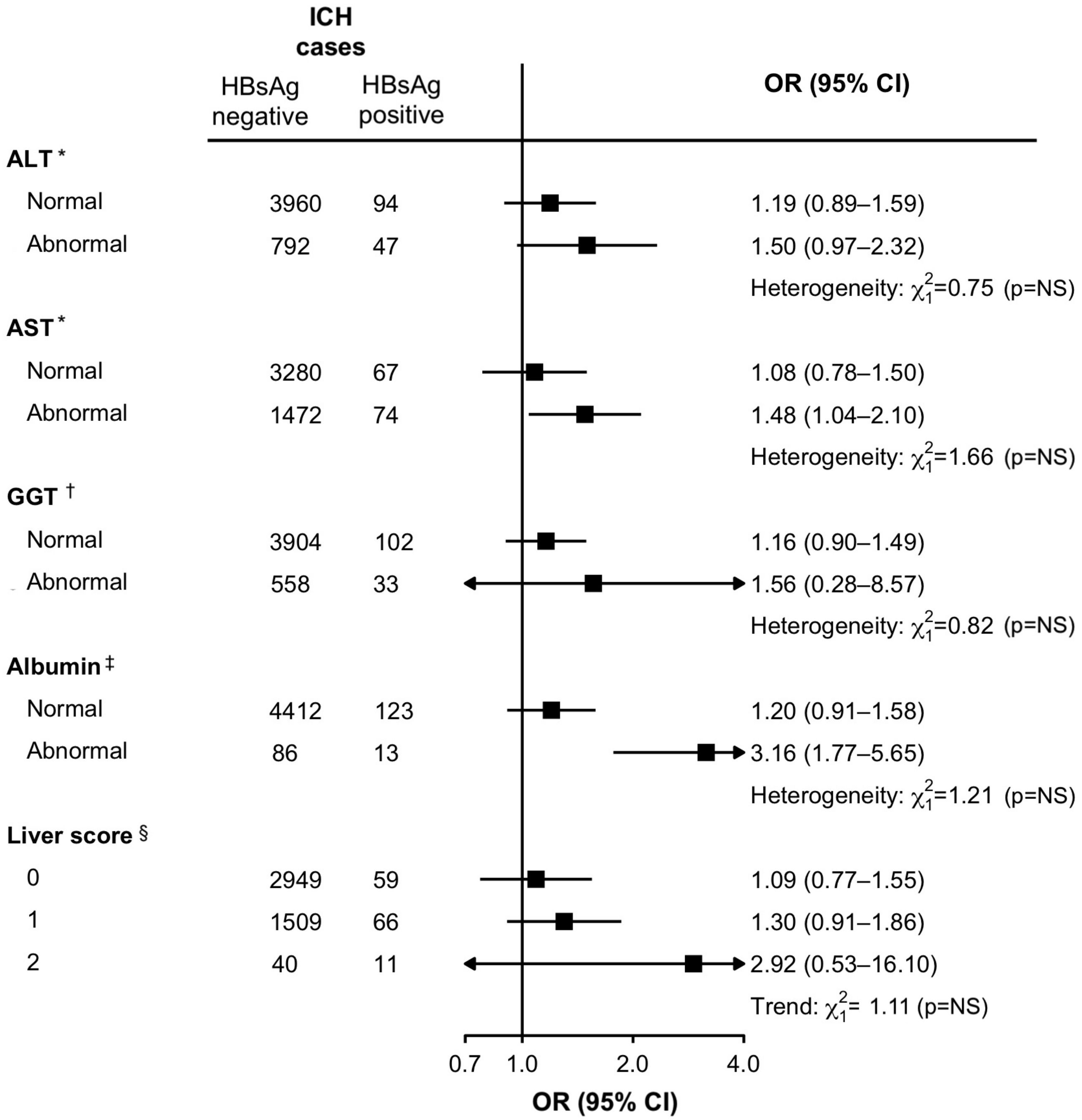

*Abnormal >30 µmol/L; † Abnormal >50 µmol/L; ‡ Abnormal <35g/L; § one point awarded for abnormal AST or ALT, and for abnormal albumin. Estimates adjusted for age (5-year categories), sex and study site where possible. Abbreviations: HBsAg, hepatitis B surface antigen; OR, odds ratio; ALT, alanine transaminase; AST, aspartate aminotransferase; GGT, Gamma-glutamyl transferase.
